# Stereo- and regioselectivity of the hetero-Diels–Alder reaction of nitroso derivatives with conjugated dienes

**DOI:** 10.3762/bjoc.12.184

**Published:** 2016-09-01

**Authors:** Lucie Brulíková, Aidan Harrison, Marvin J Miller, Jan Hlaváč

**Affiliations:** 1 Department of Organic Chemistry, Faculty of Science, Palacký University, 17. Listopadu 12, 77146 Olomouc, Czech Republic; 2 Department of Medicinal Chemistry, Institute of Molecular and Translational Medicine, Hněvotínská 5, 77900 Olomouc, Czech Republic; 3 Department of Chemistry and Biochemistry, University of Notre Dame, Notre Dame, IN 46556, USA

**Keywords:** diene, hetero-Diels–Alder, nitroso compounds, regioselectivity, stereoselectivity

## Abstract

The hetero-Diels–Alder reaction between a nitroso dienophile and a conjugated diene to give the 3,6-dihydro-2*H*-1,2-oxazine scaffold is useful for the synthesis of many biologically interesting molecules due to the diverse opportunities created by subsequent transformations of the resulting 1,2-oxazine ring. This review discusses the rationale for the observed regio- and stereoselectivity and the methods developed in recent years used to control and improve the stereo- and regioselectivity for the synthesis of 1,2-oxazine scaffolds.

## Review

### Introduction

The hetero-Diels–Alder reaction represents one of the most important methods in organic synthesis, providing various biologically active compounds. It is a variant of the Diels–Alder reaction where either the diene or the dienophile contains a heteroatom. Hetero-Diels–Alder reactions between conjugated dienes and nitroso dienophiles affording 1,2-oxazines are utilized for the synthesis of many biologically active molecules and natural products as well as for the mild functionalization and derivatization of diene-containing natural products [1–10]. The reversibility of this reaction plays a considerable role in both the observed regio- and stereocontrol of the nitroso Diels–Alder reaction, requiring a detailed examination of the kinetic versus thermodynamic effects.

**Scheme 1:**
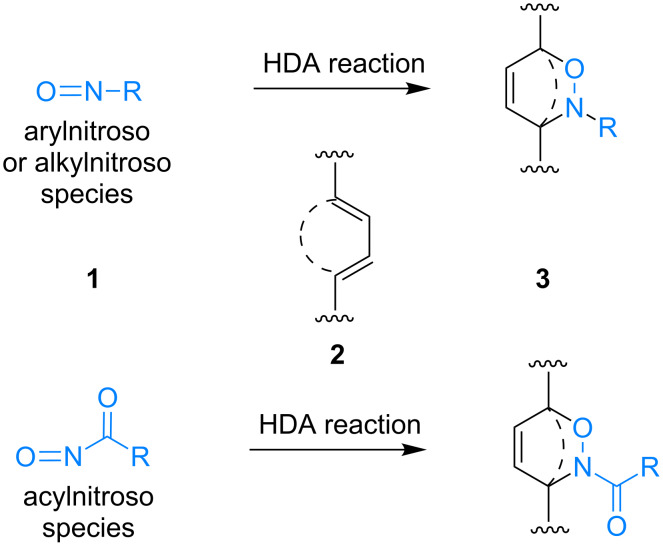
Nitroso hetero-Diels–Alder reaction.

The nitroso hetero-Diels–Alder reaction involves the formation of the 3,6-dihydro-2*H*-1,2-oxazine scaffold **3** from nitroso dienophiles **1** and dienes **2** in a [4 + 2] cycloaddition reaction (Scheme 1).

**Scheme 2:**
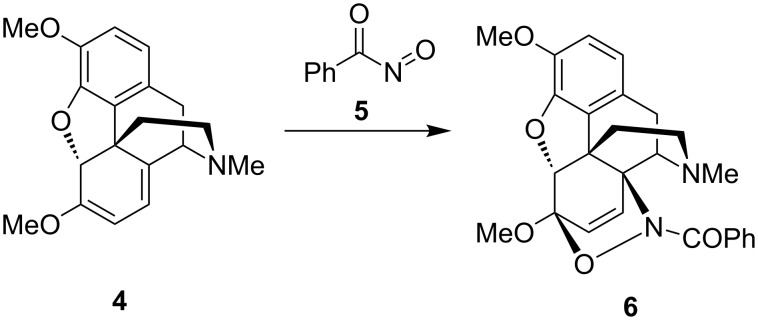
The hetero-Diels–Alder reaction between thebaine (**4**) and an acylnitroso dienophile **5**.

The first nitroso hetero-Diels–Alder reactions using alkyl- or arylnitroso dienophiles were reported by Wichterle [11] and Arbuzov [12–13] in 1947 and 1948, respectively. Among the early reported reactions using an acylnitroso compound as the dienophile, an interesting study by Kirby and Sweeny in 1973 [14] reported that the acylnitroso dienophile **5** was generated in the presence of thebaine (**4**) to selectively give the 1,2-oxazine **6** (Scheme 2).

Several excellent reviews on nitroso hetero-Diels–Alder reactions have been published in the past, including general reviews on hetero-Diels–Alder reactions and their applications in organic synthesis [15–24], applications of nitroso hetero-Diels–Alder reactions for the synthesis of azasugars [10], and the utilization of nitroso hetero-Diels–Alder reactions in natural product synthesis [9,25] and the synthesis of bioactive molecules [26]. However, none of these reviews focused on general aspects of the regio- and stereoselectivity of the nitroso hetero-Diels–Alder reaction and the possibility of its control. These aspects were partially covered in a review by Yamamoto in 2006 [27], which focused on asymmetric nitroso hetero-Diels–Alder reactions, following the review by Miller that was published in 1998 [28]. The possibility to control nitroso HAD reactions is the most relevant fact for using the nitroso hetero-Diels–Alder reaction for the syntheses of biologically important molecules.

This review will thus focus on the regio- and stereoselectivity of the nitroso hetero-Diels–Alder reaction. Special emphasis is drawn to the influence and control of the reaction under solution and solid-state reaction conditions. The main aim of this review is to provide insight into the fundamental relationships between the structures of the reactants and the regio- and stereoselectivity of the hetero-Diels–Alder reaction as well as the possibility of controlling the regio- and stereoselectivity of 3,6-dihydro-2*H*-1,2-oxazine formation.

#### Nitroso compounds and dienes

**Figure 1:**
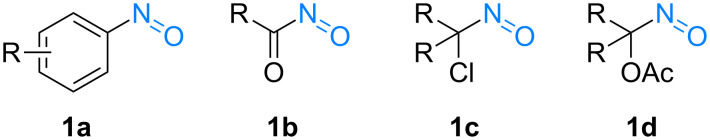
Examples of nitroso dienophiles frequently used in hetero-Diels–Alder reaction studies.

**Nitroso compounds:** Nitroso compounds are highly reactive dienophiles often used for the hetero-Diels–Alder reaction [16,27–29] and the most frequently used representatives are depicted in Figure 1. Compounds **1a–c** will be studied in more detail in this review.

**Scheme 3:**
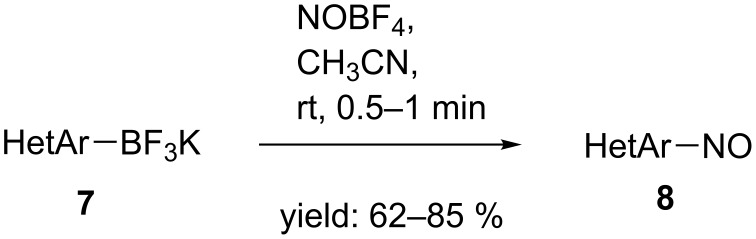
Synthesis of arylnitroso species by substitution of a trifluoroborate group [36].

Arylnitroso compounds are, in comparison to acylnitroso analogues, quite stable, and in many cases, they can be isolated and stored. An extensive review by Gowenlock and Richter-Addo from 2004 [29] describes methods for the preparation of arylnitroso agents including the direct substitution of various functionalities and the transformation of pendant groups. The direct substitution of a H atom [30–32] or a metal substituent [33–35] on the aromatic ring was recently extended to the substitution of trifluoroborate groups in aromatic systems by nitrosotetrafluoroborate. The reaction affords good yields for both electron-withdrawing and donating substituents and works even in heteroaromatic systems (HetAr, **7**) (Scheme 3) [36].

**Scheme 4:**
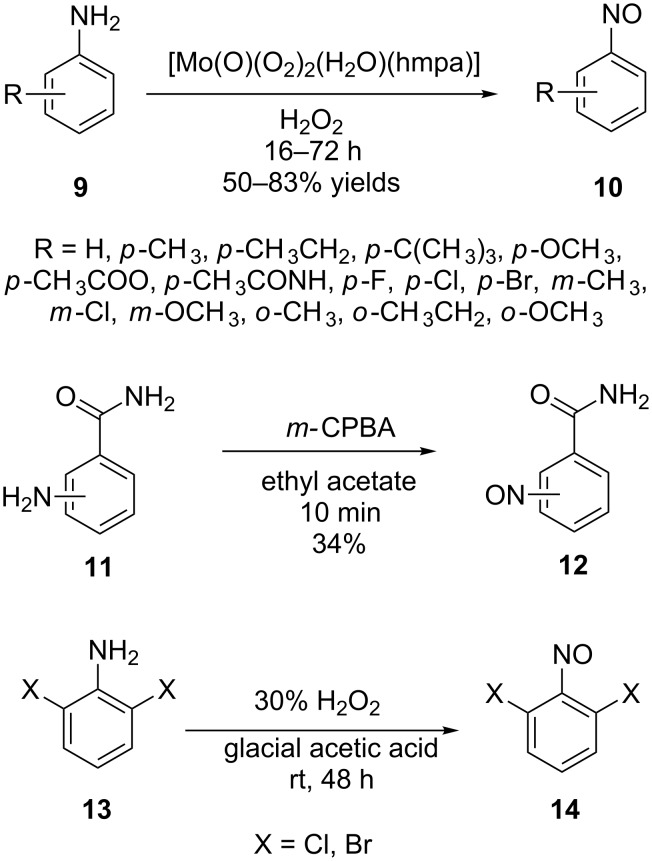
Synthesis of arylnitroso compounds by amine oxidation.

**Scheme 5:**
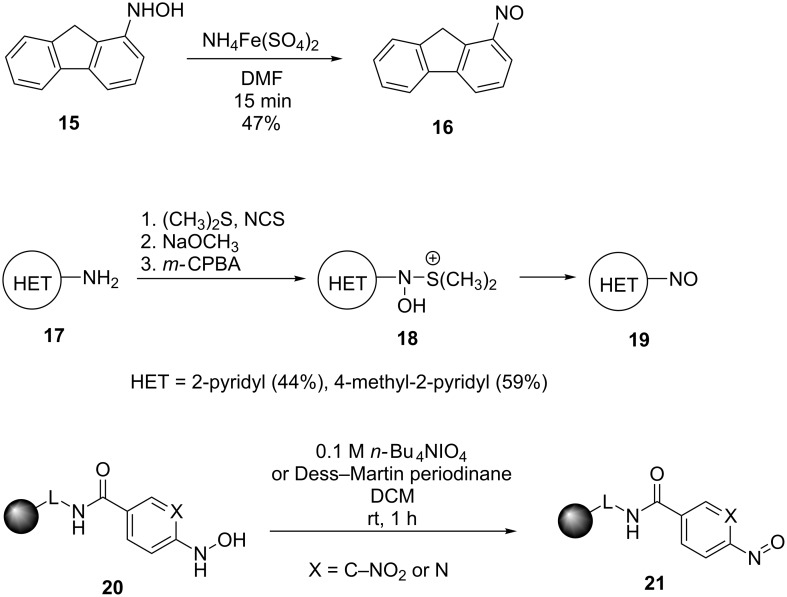
Synthesis of arylnitroso compounds by hydroxylamine oxidation.

The introduction of the nitroso group through the transformation of pendant functional groups includes the oxidation of primary amines [37–41] (Scheme 4) and hydroxamic acids [42–44] (Scheme 5) and the reduction of nitro compounds [45–47]. As oxidants for the amino group transformation, hydrogen peroxide and *m*-CPBA are the most popular (see examples in Scheme 4).

In the literature, the oxidation of hydroxylamines is described most frequently using Fe(III) salts, *m*-CPBA or TBAPI and the reaction is performed exclusively using a solid-phase synthetic approach (see examples in Scheme 5).

More recently, the method was extended to the mild copper-catalyzed aerobic oxidation of hydroxylamines [48–50]. In 2014, the Lykakis group reported the selective oxidation of various arylamines into the corresponding nitrosoarenes through polyoxometalate anions supported on mesoporous TiO_2_ nanoparticle assemblies using H_2_O_2_ [51].

**Scheme 6:**
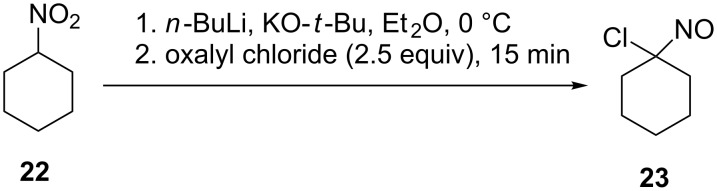
Synthesis of chloronitroso compounds by the treatment of a nitronate anion with oxalyl chloride.

Geminal chloronitroso compounds are synthesized or in situ generated from their corresponding oximes by chlorination. As halogen source elemental chlorine [52–54], nitrosyl chloride [55], alkyl hypochlorites [56], *N*-chlorourea [57], *tert*-butyl hypochlorite [58] or related electrophilic halogen precursors [59–60] may be used. However, most of these methods result in the formation of nitro derivatives along with the desired nitroso compounds. Relatively new methods for the conversion of nitroalkanes into geminal chloronitroso compounds involving treatment of a nitronate anion with oxalyl chloride were recently published [61–62] (Scheme 6).

**Scheme 7:**
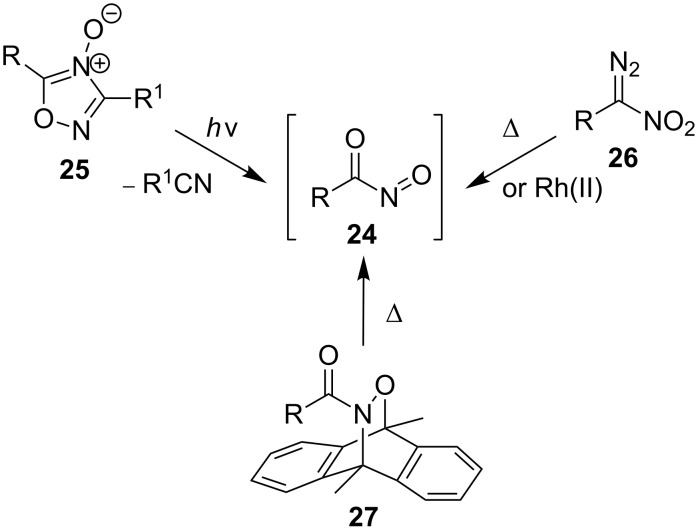
Non-oxidative routes to acylnitroso species.

Acylnitroso compounds are generally prepared and used in situ due to their extremely reactive nature and the conditions have been summarized previously [16]. These include the oxidation of nitrile oxides [63] or the corresponding hydroxamic acids using, for example, periodate [14], Dess–Martin periodinane [64], Swern oxidation conditions [65], lead and silver oxide [66], and transition-metal oxidation with peroxide as the oxidant [67]. In a recent work by Tusun dirhodium caprolactamate [68], and the aerobic oxidation in the presence of catalytic amounts of Cu(II) and pyridine [48–50] were used for the preparation of acylnitroso compounds. In 2015, the Whiting group reported an extensive study of acylnitroso compounds prepared in situ by the catalytic aerobic oxidation of hydroxycarbamate using CuCl_2_ and 2-ethyl-2-oxazoline in methanol [69]. Additionally, acylnitroso compounds can be generated by the rearrangement of diazonitroalkanes **26** [70], the photochemical cleavage of 1,2,4-oxadiazole-4-oxides **25** [71] and the cycloreversion of 9,10-dimethylantracene adducts **27** (Scheme 7) [72–73].

**Dienes:** There is a wide range of acyclic and cyclic dienes available for the nitroso hetero-Diels–Alder reaction. Cyclic dienes such as cyclopenta-, cyclohexa- and cycloheptadiene as well as a number of more complex substituted derivatives have been reported as being highly reactive substrates for the reaction [2,74–77]. Acyclic dienes are still reactive even while bearing a number of substituents, including both electron-donating and withdrawing groups at the 1 and/or 2 positions. This compatibility with such a range of dienes reflects the remarkable inherent activity of the nitroso agents, especially the acylnitroso moieties.

The reactivity of dienes has also been studied for the reaction with acyl- [78] and arylnitroso dienophiles [42] that are bound to a solid support. In general, cyclic dienes were more reactive than their acyclic counterparts, and dienes with electron-donating substituents, such as α-terpinene and 2,4-hexadien-1-ol, were more reactive than those with electron-withdrawing substituents such as sorbic acid or ethyl sorbate and their unsubstituted counterparts, 1,3-cyclohexadiene and 2,4-hexadiene. However, the electronic effect is not the only factor responsible for the ease of the nitroso hetero-Diels–Alder reaction. In the reaction of arylnitroso dienophiles containing a nitro group in close proximity to the reaction center, unsubstituted dienes show a higher reactivity than substituted dienes, due to steric effects.

#### Mechanistic studies of the nitroso hetero-Diels–Alder reaction

**Figure 2:**
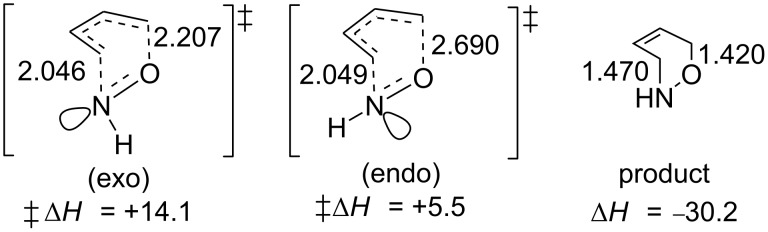
RB3LYP/6-31G* computed energies (in kcal·mol^−1^) and bond lengths for *exo* and *endo*-transition states and the product of the nitroso hetero-Diels–Alder reaction between HNO and 1,3-butadiene.

Pioneering computational studies on the mechanism of the intermolecular nitroso hetero-Diels–Alder reaction by Houk [79–80] demonstrated that the reaction proceeds in a concerted fashion through an asynchronous transition state. In the two calculated transition states (endo and exo), the ratio of the distance between C–O to that between C–N was more than one, whereas in the product, this was reversed (Figure 2). Using RB3LYP/6-31G*//RB3LYP/6-31G* theory, for the model reaction between HNO and butadiene, the favored *endo*-transition state activation energy was found to be 8.6 kcal/mol lower than for the *exo* state. When a number of substituted nitroso compounds were subsequently investigated, the *endo*-transition state persisted in having a lower activation energy for all compounds tested, relative to the *exo*. This preference is due to the “*exo* lone pair effect” resulting from the repulsion between the nitrogen lone pair of electrons and the π electrons of the electron-rich diene in the *exo*-transition state (Figure 2).

**Scheme 8:**
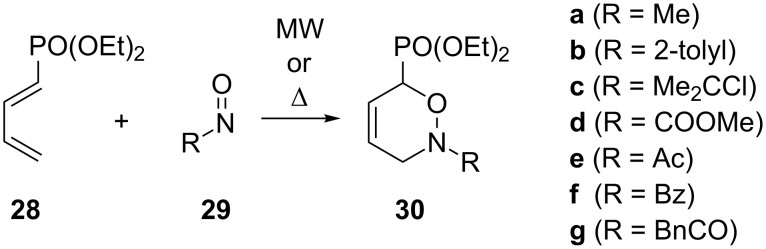
Hetero-Diels–Alder cycloadditions of diene **28** and nitroso dienophiles **29**.

Beyond Houk’s studies, other groups also have reported computational investigations of hetero-Diels–Alder reactions in the last decade, e.g., [21,69,81–87]. In 2010, the Marchand–Brynaert group published several results of asymmetric hetero-Diels–Alder reactions of phosphonyl-1,3-butadienes with various nitroso dienophiles [21,85–87]. A computational investigation of the hetero-Diels–Alder reactions of 1-diethoxyphosphonyl-1,3-butadiene (**28**) with various nitroso heterodienophiles **29** (Scheme 8) was determined at the B3LYP/6-31G** level [86]. All calculations were performed with density functional theory (DFT) using the B3LYP functional implemented in the Gaussian 98 software. Nitrosomethane (**29a**) was selected as a model heterodienophile for a preliminary investigation. It was calculated that the hetero-Diels–Alder reaction between **29a** and 1-diethoxyphosphonyl-1,3-butadiene (**28**) proceeds as a polar cycloaddition via a two-stage process involved in one step, in which the C4–N5 centers’ interaction governs the reaction [83]. The phosphonate moiety also drives the reaction towards one regioisomer because of the activating effect and steric hindrance of the phosphonate substituent onto the butadienyl moiety.

**Figure 3:**
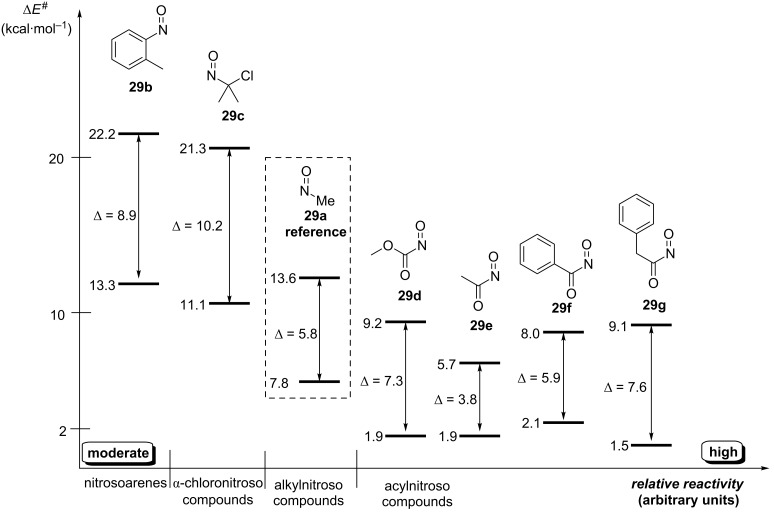
Relative reactivity (Δ*E*^#^) and regioselectivity (Δ) for hetero-Diels–Alder of **28** and nitroso dienophiles **29a–g**. Arrows indicate the Δ*E*^#^ between two regioisomers.

The computed activation barriers for the cycloaddition of **28** to some representative nitroso compounds **29** were used to elucidate the structure–reactivity relationships and to predict the regioselectivity. The results indicated that the nitroso dienophiles’ reactivity towards diene **28** increases from nitrosotoluene **29b** and α-chloronitroso compound **29c** to acylnitroso compounds **29d**–**g** (Figure 3).

In the same year, the Marchand–Brynaert group reported computational and experimental studies on the hetero-Diels–Alder reaction of the chiral 1-phosphono-1,3-butadiene **31** with nitroso dienophiles **32** (Scheme 6) [87]. The authors studied the reactivity of chiral 1-phosphonodienes modified with the bicyclic (*R,R*)-1,3,2-dioxaphospholane **31a** or (*R*,*R*)-1,3,2-diazaphospholidine **31b–e** diene auxiliaries to model nitroso dienophiles **32**.

**Scheme 9:**
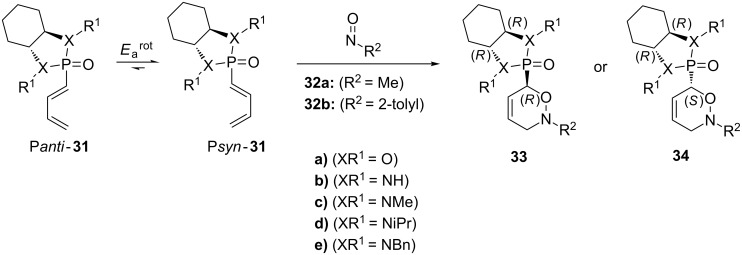
Reaction of chiral 1-phosphono-1,3-butadiene **31** with nitroso dienophiles **32**.

Within the series of dienes, **31e** showed the highest computed stereoselectivity (Δ*E*_a_^sel^ = 3.1–3.7 kcal·mol^−1^). However, the experimental reaction of **31e** with nitroso derivative **32b** resulted in a 1:1 mixture of diastereomers **33** and **34**. The authors explained this observation by a low-energy discrimination between the P*anti* and P*syn* conformers of chiral diene **31e** (Scheme 9) and by the high asynchronicity of the formation of the new bond with a nitroso partner (P*anti* and P*syn* refers to the conformation of the O=P–C(1)–C(2) dihedral angles).

**Scheme 10:**
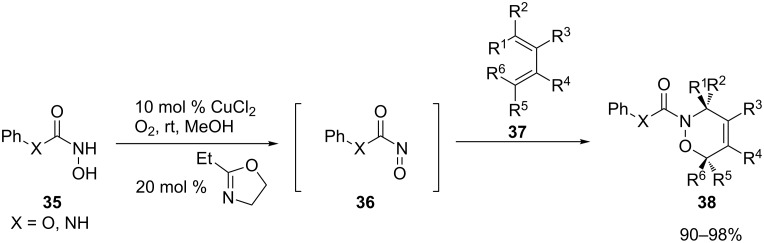
Hetero-Diels–Alder reactions of hydroxamic acids **35** with various dienes **37**.

Recent research of the Whiting group revealed a wide spectrum of results on the reactions of hydroxamic acid analogues **35** with various dienes **37** using the copper/oxazoline/air catalytic system (Scheme 10) [69]. Conclusions of an experimental as well as a computational approach to understanding the reactions of acylnitroso compounds in [4 + 2] cycloadditions indicate several facts. The copper–oxazoline complex behaves as an excellent catalyst for the aerobic oxidation of acylhydroxamic acids. However, this system is useful only for hydroxamic acids containing a heteroatom between the aryl and carbonyl group. Further, the yields of products varied from high to moderate, depending upon the reaction time due to the competitive decomposition of the nitroso species, reducing the yield. The chemoselectivity of this system also depends on the reactivity of the hydroxamic acid: the higher the reactivity, the lower the chemoselectivity. Moreover, DFT calculations of this type of reaction confirmed the preference for *endo* transition states. These calculations showed that the acylnitroso species are superreactive, and the activation energies are lower than the isomerization barriers between some *cis* and *trans*-butadienes.

### Regioselectivity of the nitroso hetero-Diels–Alder reaction

Because two regioisomeric adducts can be formed when an unsymmetrical diene reacts with a nitroso species, the regioselectivity is an important issue of nitroso hetero-Diels–Alder reactions. Thus, the regioselectivity of this reaction was studied by several groups, e.g., [79–80,84,86,88–90], and it was confirmed to be highly dependent on several factors. These are for example the number and nature of substituents on the diene and the dienophile, and the reaction conditions such as temperature, pressure, solvent, and presence of catalyst, etc.

Further, the regioselectivity can be influenced by the reversible nature of the hetero-Diels–Alder reaction (i.e., thermodynamic control). This was demonstrated by Miller in both higher temperature and Cu(I)-mediated hetero-Diels–Alder reactions of a chiral ligand with nitroso-heteroarene reactants [91]. Nicholas and co-workers investigated the thermal (uncatalyzed) and Cu(I)-catalyzed reactions of 2-nitrosopyridine with various dienes [92].

**Scheme 11:**
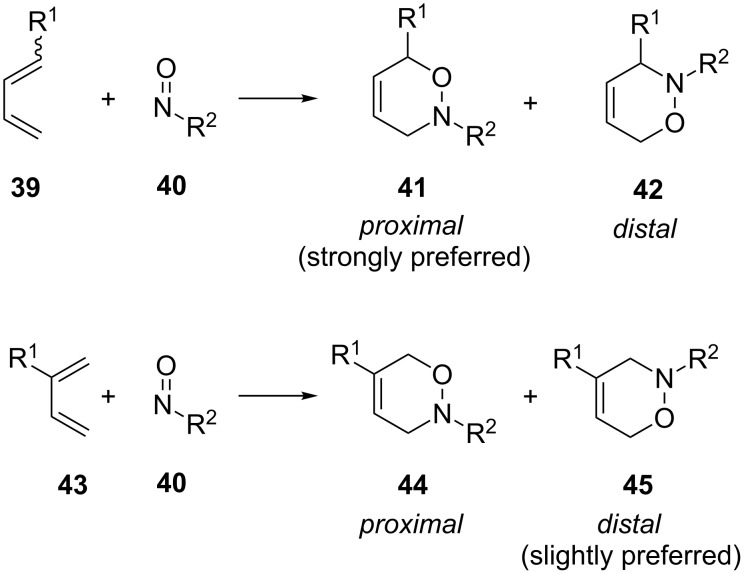
General regioselectivity of the nitroso hetero-Diels–Alder reaction observed with unsymmetrical dienes.

Generally, the cycloaddition of an unsymmetrical diene and nitroso compound will lead to two regioisomers – *proximal* and *distal* (Scheme 11). The terms *proximal* and *distal* were first used by Boger, who defined them as follows: "*Proximal* and *distal* refer to the relative orientation (distance) of the dienophile center of highest priority (nitroso oxygen) with the diene center of highest priority (substituted center of cyclohexadiene)" [93]. Computational results [79] showed that for 1-substituted dienes **39**, the *proximal* isomer **41** should be strongly preferred due to the interaction between the HOMO at the C4 position of the diene and the LUMO of the nitroso nitrogen. In case of 2-substituted dienes **43**, the *distal* isomer **45** should be slightly preferred due to the interaction between the nitroso nitrogen LUMO and the HOMO at the C1 position.

These interactions can be further influenced by substituents on both the nitroso dienophile and the diene. The HOMO on 1-substituted dienes should be the highest at the 4-position. Because the LUMO of the nitroso derivative is the largest at the nitrogen, this should favor the proximal regioisomer. Therefore, electron-donating substituents on the dienophile should be most effective in this way, followed by conjugating substituents and electron-withdrawing groups. For the diene, electron-withdrawing substituents decrease the energy of the LUMO and thereby increase the interaction with the nitrogen HOMO to further increase the preponderance of the *proximal* isomer for 1-substituted dienes and the *distal* isomer for 2-substituted dienes. Overall, substituents that are strongly electron withdrawing or donating have a more pronounced effect than those that are not as strong. The only change to this general trend is that with electron-donating substituents, 1-substituted dienes favor the *distal* isomer, while for 2-substituted dienes, the *proximal* isomer dominates, albeit both very weakly.

This general rationale for the regioselectivity of the nitroso hetero-Diels–Alder reactions proposed by Houk [79–80] was clearly summarized by the Kouklovsky group [89] (Table 1). The regioselectivity depends on the nature of the nitroso derivative and on the nature and position of the substituents on the diene. The valuable impact on the reaction selectivity is given by the configuration of the diene.

**Table 1:** General overview of the regioselectivity of nitroso hetero-Diels–Alder reactions with different dienes.

Diene^a^	Alkyl- and arylnitroso		Acylnitroso	

	major regioisomer	selectivity level	major regioisomer	selectivity level

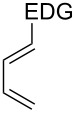	*proximal*	medium	*proximal*	high
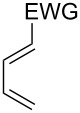	*proximal*	high	*proximal*	high
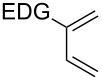	*proximal*	weak	*distal*	medium
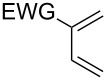	*distal*	medium	*distal*	weak
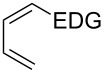	*distal*	weak	–	–
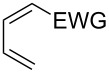	*proximal*	high	–	–

^a^EDG = electron-donating group, EWG = electron-withdrawing group.

**Scheme 12:**
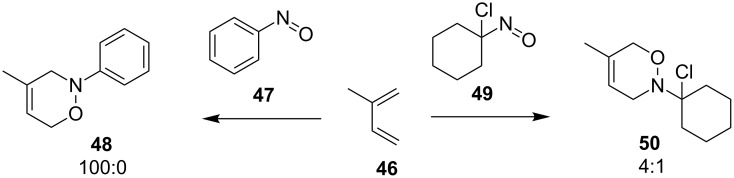
Effect of the nitroso species on the regioselectivity for weakly directing 2-substituted dienes.

Due to the weak directing effect of the 2-substituent on the diene **46** (Scheme 12), the preference could also be altered by the nature of the dienophile [79]. This is exemplified by the reactions of the arylnitroso derivative **47** or the chloronitroso derivative **49**, which gave the *distal* isomer **48** or *proximal* isomer **50**, respectively.

**Scheme 13:**
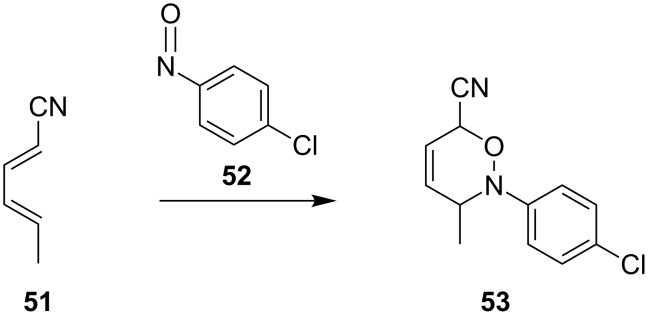
Regioselectivity of 1,4-disubstituted dienes **51**.

In disubstituted dienes, the substituent effects were found to be additive, and predictions can be made based on the relative position and strength of the electron-withdrawing or donating nature of these substituents [20,94]. This can be seen for the hetero-Diels–Alder reaction of disubstituted diene **51** with *p*-chloronitrosobenzene (**52**) to give only the *proximal* isomer **53** (Scheme 13).

**Scheme 14:**
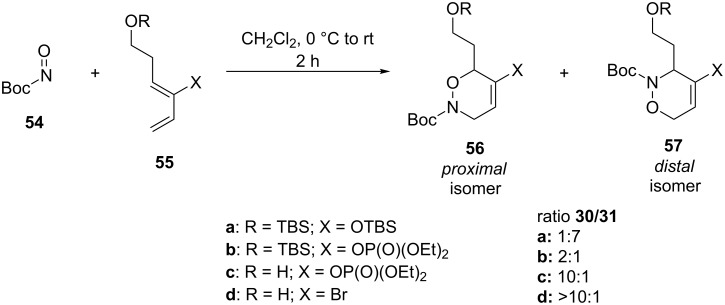
Nitroso hetero-Diels–Alder reaction between Boc-nitroso compound **54** and dienes **55**.

Recently, the Kouklovsky group reported a highly regioselective nitroso hetero-Diels–Alder cycloaddition with 1,2-disubstituted dienes, leading to the selective formation of the *proximal* isomer [89]. First, they studied the reaction of Boc-nitroso (Boc = *tert*-butoxycarbonyl) reagent **54** with different dienes **55** (Scheme 14). On basis of previous experience and Houk’s rules, the substituent at the C2 position should favor the *distal* isomer, whereas the side chain on the C1 position should strongly favor the formation of the *proximal* isomer. The observed selectivities showed that for the reaction of dienes with substituents **a**, mainly the *distal* isomer **57** is obtained, while reactions of substrates **b**,**c** gave mainly the *proximal* isomers **56**. Finally, the authors reported that the optimal conditions for a regioselective nitroso hetero-Diels–Alder reaction with a 1,2-disubsituted diene are affected by several features: a bulky substituent at C1 and an electron-donating group at C2 provided the *distal* isomer, while a nonbulky substituent at C1 and an electron-withdrawing group at C2 gave the *proximal* isomer.

**Scheme 15:**
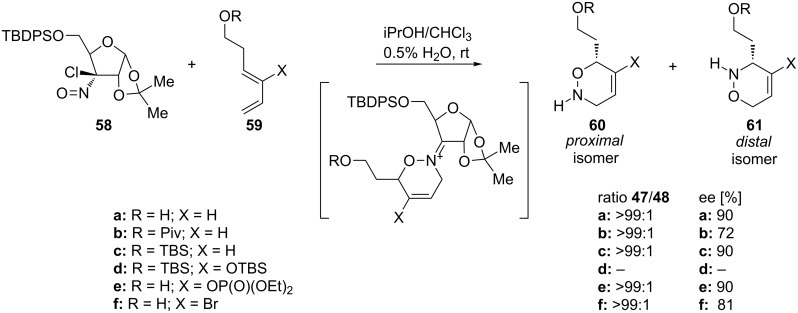
Nitroso hetero-Diels–Alder reaction between Wightman reagent **58** and dienes **59**.

Moreover, they applied the rules for regioselectivity to alkylnitroso compounds – Wightman chloronitroso reagent **58** – for which a complete regioselectivity was observed (Scheme 15). The reaction also proceeded with high stereoselectivity because of the presence of a chiral nitroso agent.

**Scheme 16:**
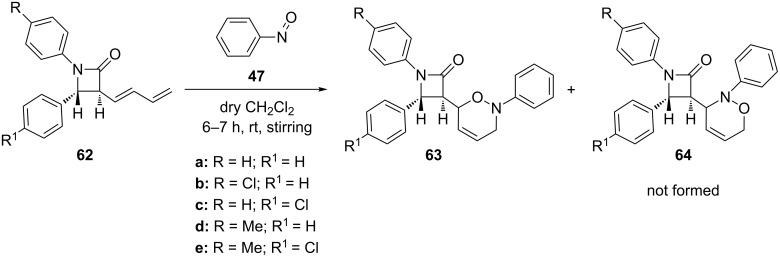
Regioselective reaction of 3-dienyl-2-azetidinones **62** with nitrosobenzene (**47**).

The regioselective nitroso hetero-Diels–Alder cycloaddition was also observed during the reaction of 3-dienyl-2-azetidinones **62** with nitrosobenzene (**47**), specifically providing 1,2-oxazine-substituted β-lactams **63** (Scheme 16) [95], which is in accordance with the general prediction. The exclusive regioselectivity of this reaction is due to steric effects.

**Scheme 17:**
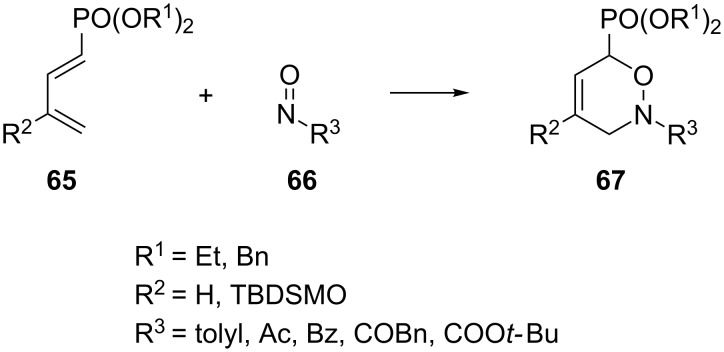
The regioselective reaction of 1,3-butadienes **65** with various nitroso heterodienophiles **66**.

Similar conclusions result from the reaction of 1,3-butadienes **65** with various nitroso heterodienophiles **66**, giving proximal isomer **67** (Scheme 17) [85].

**Scheme 18:**
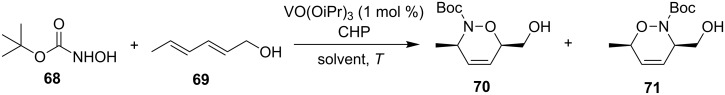
Catalysis of the nitroso hetero-Diels–Alder reaction by vanadium in the presence of the oxidant CHP (cumyl hydroperoxide).

Recently, a vanadium-catalyzed nitroso hetero-Diels–Alder reaction between hexa-2,4-dien-1-ol (**69**) and Boc-protected hydroxylamine **68** was reported by Hoshino [96] (Scheme 18). This reaction, with the hydroxylamine oxidized in situ to the nitroso dienophile in the presence of vanadium in different solvents (CH_2_Cl_2_ or toluene) and at different temperatures (−20 °C or rt), gave the hetero-Diels–Alder products **70**,**71**, with the ratio of **70** and **71** varying from 71:29–83:17, and yields between 65 and 99%.

#### Comparison of the regioselectivity in solution and solid-phase nitroso hetero-Diels–Alder reactions

**Figure 4:**
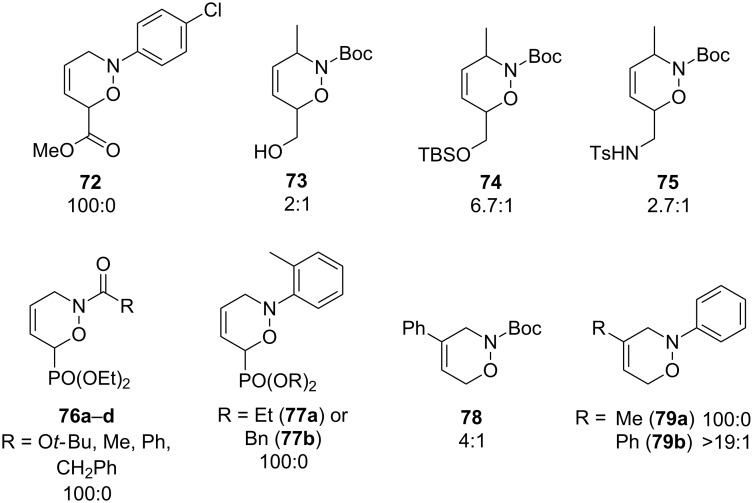
1,2-Oxazines synthesized in solution with moderate to high regioselectivity, showing the favored regioisomer in each case.

Experimentally, the general rules of regioselectivity mentioned above hold true in most cases for both solution and solid-phase hetero-Diels–Alder reactions. The regioselectivity of the nitroso hetero-Diels–Alder reaction in solution has been studied in detail, e.g., [79–80,87,97], and the general rules for regioselectivity were shown to be valid for a number of simple hetero-Diels–Alder products **72**–**79** (Figure 4).

**Figure 5:**
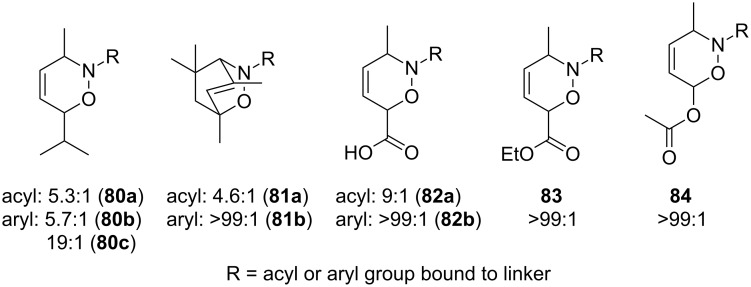
1,2-Oxazines synthesized in the solid phase with moderate to high regioselectivity, showing the favored isomer in each case.

The hetero-Diels–Alder reaction in the solid phase has been studied to a far lesser degree. However, an analysis of the available data for a number of 1,2-oxazines **80**–**84** indicates, that, in most cases, the same rules as for solution-phase chemistry are valid whether the *proximal* or *distal* regioisomer is favored (Figure 5) [42,78].

**Scheme 19:**
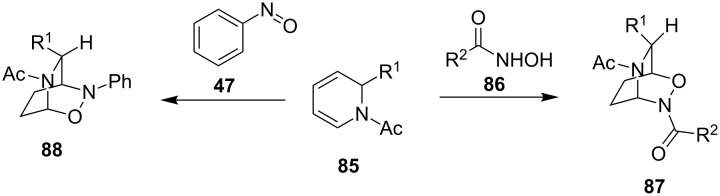
Regioselectivity of solution-phase nitroso hetero-Diels–Alder reaction with acyl and aryl nitroso dienophiles.

In most cases, the regioselectivity of the cycloadditions in both the solid phase and in solution is the same for acyl and arylnitroso dienophiles. However, in some cases, the selectivity reverses or the performance of the reaction and selectivity drastically alter. In solution, the reaction of *N*-acyl-1,2-dihydropyridines **85** with nitrosobenzene (**47**) yielded cycloadducts **88**. On the other hand, the reactions with acylnitroso dienophiles **86** afforded the reversed regioisomeric cycloadducts **87** (Scheme 19) [98–99].

**Scheme 20:**
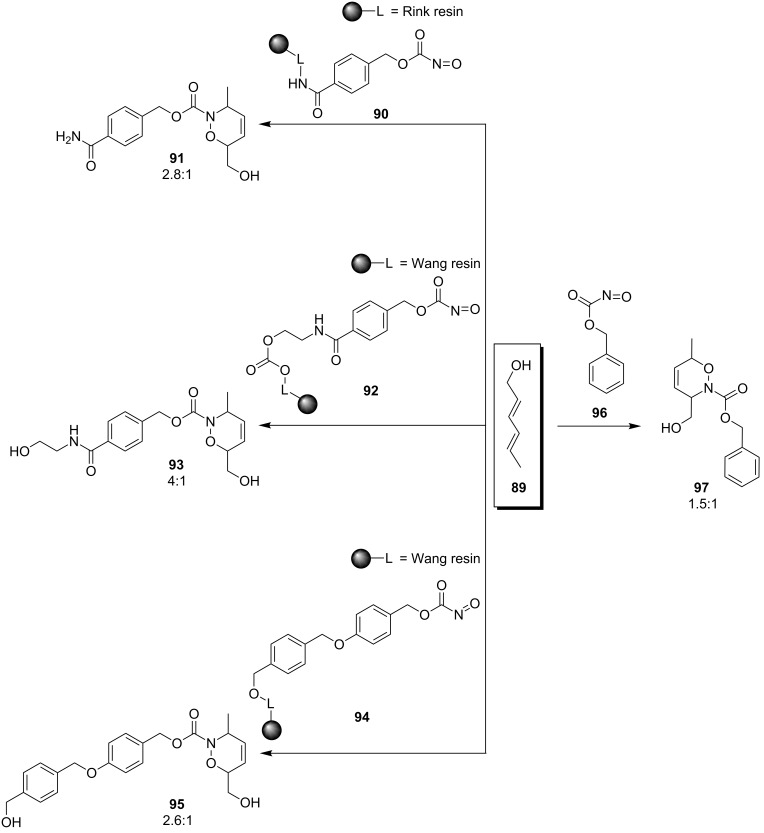
Favored regioisomeric outcome for the solution and solid-phase reactions, giving hetero-Diels–Alder products **91**, **93**, **95** and **97**.

A comparison of the differences in selectivity for the same diene reacting with an acylnitroso dienophile in the solid phase and in solution is difficult because of a lack of reported reactions using the same acylnitroso moiety. Scheme 20 shows the difference in the regioselectivity of carbamoylbenzyl 1,2-oxazines prepared from diene **89** and benzyl nitrosoformate (**96**) in solution (derivative **97**) [100] and 4-substituted benzyl nitrosoformates on a solid support (derivatives **91**–**95**) [78]. It is obvious that although the substitution on benzyl nitrosoformate is quite distant from the site of the 1,2-oxazine formation, it influences the regioselectivity of the reaction. Therefore, the comparison of reactions performed in solution and on a solid support is impossible.

**Figure 6:**
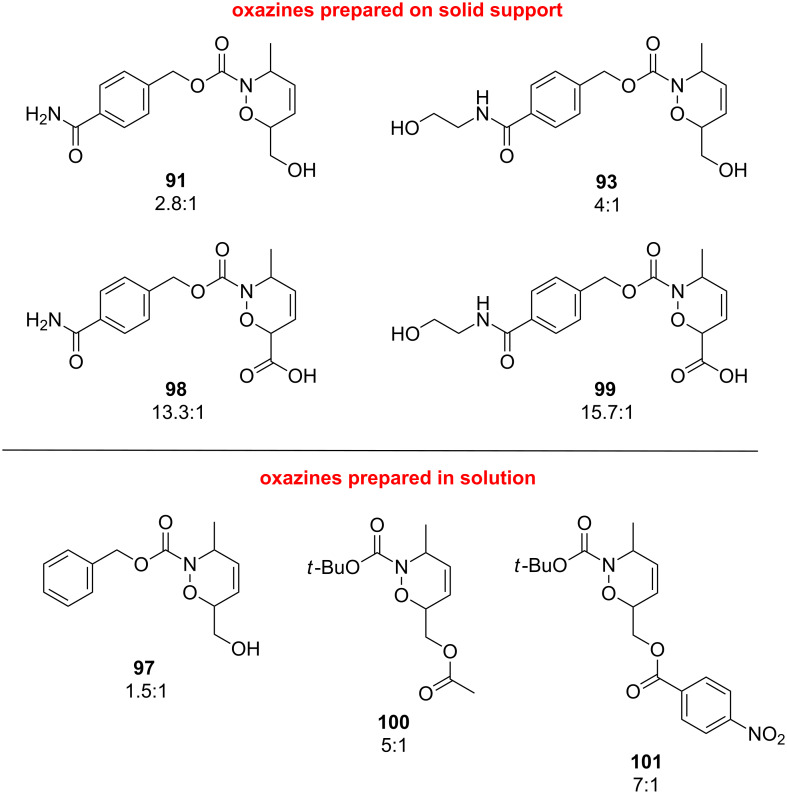
Favored regioisomers and regioisomeric ratios for 1,2-oxazines synthesized in solid phase (**91**, **93**, **98**, **99**) and in solution (**97**, **100**, **101**).

It is important to note that in the solid as well as in the solution-phase reactions, the solvent was dichloromethane throughout. When the reaction in solution shown in Scheme 20, giving 1,2-oxazine **97**, was performed in MeOH, the ratio of regioisomers was changed from 3:2 (in DCM) to 3:1 (in MeOH) [100]. The comparison between the solution and solid-phase syntheses is possible to do with respect to the substitution-regioselectivity relationship. Both in solution [100] and in the solid phase [78], the regioselectivity of the reaction could be increased by utilizing a more electronegative substituent in the diene such as sorbic acid (**98** and **99**) or an ester of sorbic alcohol (**100** and **101**, Figure 6).

#### Regiocontrol of the nitroso hetero-Diels–Alder reaction

As discussed above, the nitroso hetero-Diels–Alder reaction can be highly regioselective due to the influences of diene substituents and electronic effects. However, reports on the specific regiocontrol of the reaction are rare. In general, the regioselectivity can be enhanced by the use of asymmetric catalysis or chiral substrates. In addition, there are reported alterations to the diene, dienophile, or reaction conditions that are able to improve the regioselectivity for a given reaction.

In a solution-phase nitroso hetero-Diels–Alder reaction between an arylnitroso dienophile and an iron-bound substituted 1,3-cyclohexadiene **103**, changing the aryl ring (Ar’) from pyridine to phenyl increased the regioselectivity from 2:1 (**105**/**104**) to complete regioselectivity for the reverse isomer **104** (Table 2) [101]. The results for the regiocontrol for aryl derivatives **c** and **d** are also collected in Table 2.

**Table 2:** Regiocontrol of the reaction between an arylnitroso dienophile and an iron-bound 1,3-cyclohexadiene derivative **103**.

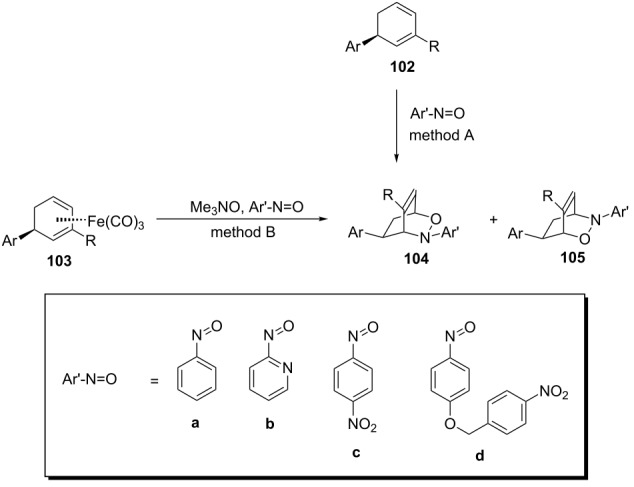					

Entry	Ar´	Ar	R	method	Yield

1	**a**	Ph	Me	B	68% of **104**
2	**b**	Ph	Me	B	60% of **105**/**104** (2:1)
3	**c**	H	H	A	92% of **104**
4	**c**	H	H	B	58% of **104**
5	**c**	Ph	Me	B	28% of **104**
6	**d**	H	H	A	25% of **104**
7	**d**	H	H	B	19% of **104**

**Scheme 21:**
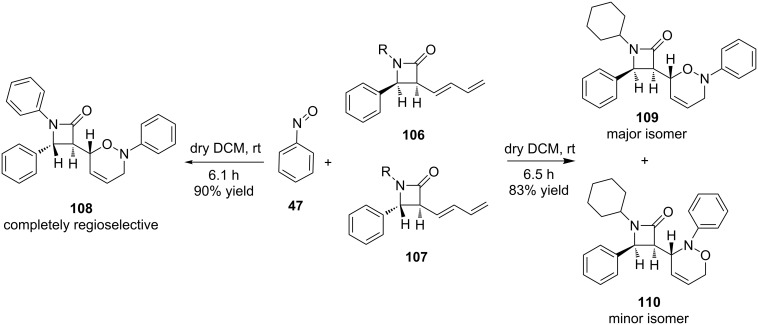
Regiocontrol of the reaction between 3-dienyl-2-azetidinones and nitrosobenzene due to change in a single stereocenter.

It was also shown that in the reaction of 3-dienyl-2-azetidinones **106** and **107** with nitrosobenzene (**47**), when the stereochemistry of one single center was altered, the regioselectivity changed. Derivative **107** reacted with nitrosobenzene (**47**) with complete regioselectivity. On the other hand, its isomer **106** gave two regioisomers in a ratio of 2:1 [88,95] (Scheme 21). This was proposed to be due to an increased steric interaction between the phenyl ring at position 4 on the β-lactam ring in **107** and the nitrosobenzene (**47**) during the formation of product **108**. For the formation of **109**, on the other hand, the steric hindrance was greatly reduced, and consequently no complete regioselectivity was observed. These results were supported by ab initio [HF/6-31G(d)] and density functional theory [B3 LYP/6-31G(d)] calculations.

**Scheme 22:**
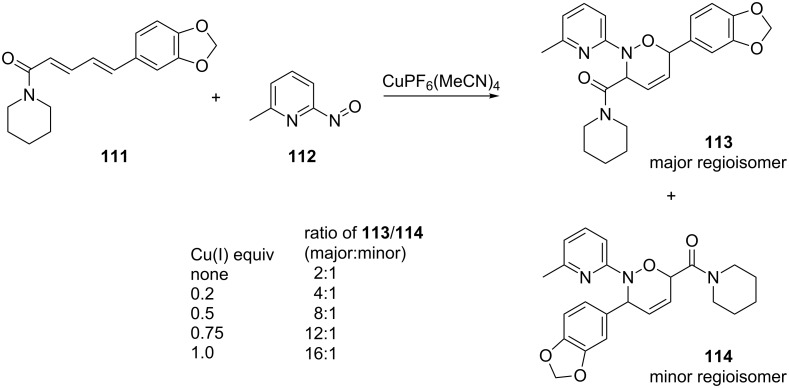
Regiocontrol of the reaction between diene **111** and 2-methyl-6-nitrosopyridine (**112**) due to metal coordination.

The use of a copper(I) species, without a complementary chiral ligand, was sufficient to alter the regioselectivity for the reaction between the piperidinyl-substituted diene **111** and 2-methyl-6-nitrosopyridine (**112**). By increasing the concentration of tetrakis(acetonitrile)copper(I)hexafluorophosphate, the regioselectivity of the major product **113** was improved from a ratio of 2:1 to 16:1 (Scheme 22) [102]. The regioselectivity of the reaction is driven by the coordination of both the nitroso dienophile and diene to the Cu(I) center of the catalytic complex, which is discussed later [103–104].

### Stereoselectivity of the hetero-Diels–Alder reaction

**Scheme 23:**
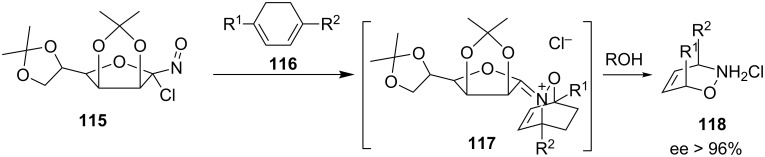
Asymmetric hetero-Diels–Alder reactions reported by Vasella [56].

Asymmetric (chiral) hetero-Diels–Alder reactions in a stereoselective (enantioselective or diastereoselective) manner have become very popular in the last decade. Pioneering works by Kresze and Vasella [56,94,105] using carbohydrate-based α-chloronitroso agents and Kirby [14] using acylnitroso compounds led to asymmetric versions of this reaction. Vasella synthesized the hetero-Diels–Alder product **118** with >96% enantiomeric excess from an α-chloro-α-nitroso ether **115**, prepared from mannose, and 1,3-cyclohexadienes **116** (Scheme 23) [56]. A similar work was reported by the Streith group in 1998 [106].

Kirby was able to show this with the reaction of the acylnitroso compound, which was generated in the presence of the optically active diene thebaine (**4**, see Scheme 2), generating the hetero-Diels–Alder product **6** with high regio- and stereoselectivity [14]. Both examples were early demonstrations of the tremendous potential of asymmetric nitroso hetero-Diels–Alder reactions, which has proven to be important in the synthesis of many biologically active molecules.

In general, there are a number of possibilities to influence the stereoselectivity of the nitroso hetero-Diels–Alder reaction, including the use of chiral dienes or dienophiles, and chiral catalysts and auxiliaries, as described below.

#### Stereoselective control of nitroso hetero-Diels–Alder reactions by the use of chiral starting materials

There are three different ways to achieve non-catalytic asymmetric hetero-Diels–Alder cycloadditions using chiral substrates: a) reaction with chiral nitroso dienophiles; b) reaction with a chiral diene; and c) reaction via double asymmetric induction, with both the diene and nitroso dienophile being chiral entities.

**Scheme 24:**
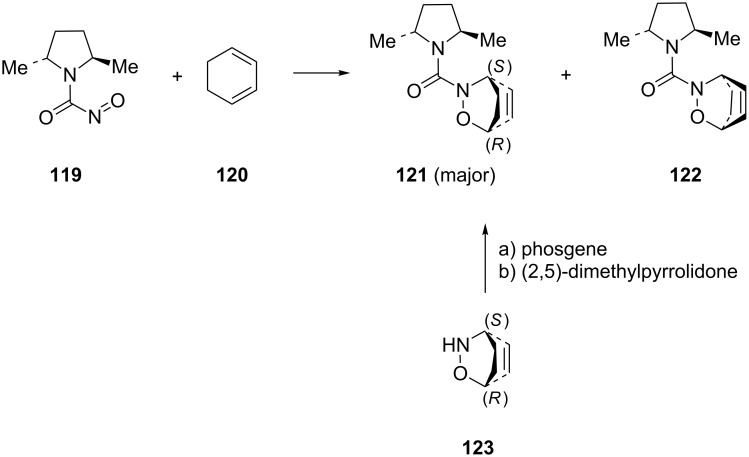
Asymmetric hetero-Diels–Alder reaction of cyclohexa-1,3-diene (**120**) with acylnitroso dienophile **119**.

**Chiral dienophiles:** The use of acylnitroso dienophiles with chiral auxiliaries has ample precedent in the literature. The application of amino acids as inducers of stereoselectivity (D- and L-*O*-methylproline or D- and L-mandelic acid) resulted in only modest stereoselectivity [107–111]. In 1996, Streith and Defoin published a successful asymmetric induction by reacting cyclohexa-1,3-diene (**120**) with the acylnitroso dienophile **119** (Scheme 24) [10]. The reaction proceeded in 81% overall yield with a major cycloadduct **121** and minor diastereomer **122**, with 98% de. The absolute configuration of the major cycloadduct was confirmed by an independent synthesis from the known bicyclic (1*R*,4*S*)-alkoxyamine **123**.

**Scheme 25:**
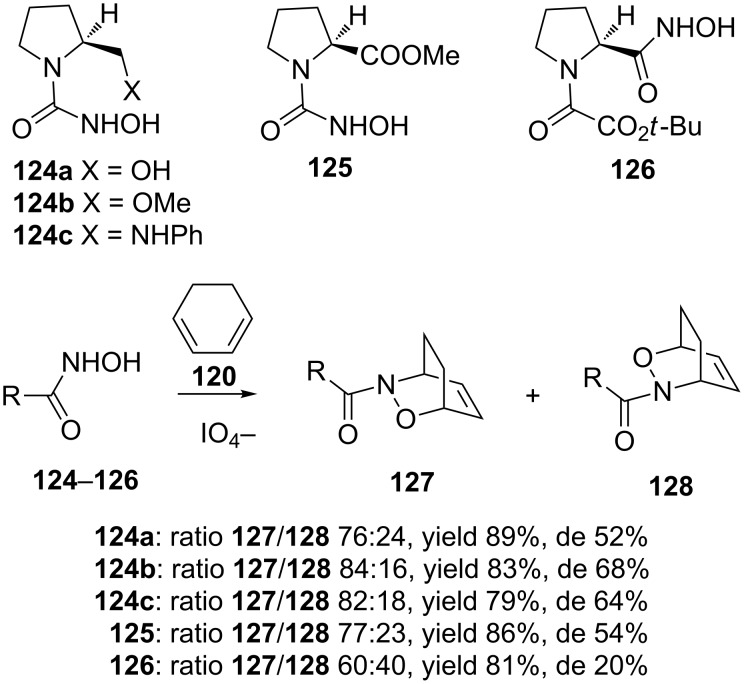
Asymmetric induction with L-proline derivatives **124**–**126**.

The reaction of the L-proline derivative **124** and its analogues **125** and **126** with cyclohexadiene (**120**) gave relatively low de values [112] (Scheme 25).

A more comprehensive study on amino acid based acylnitroso dienophiles was performed by Miller et al. [28,113]. For a number of L- and D-amino acids and derivatives **129**, including proline-derived dienophiles, the stereoselectivity of the reaction with cyclopentadiene (**130**) was tested, and again only a modest size-dependent (R^1^) stereoselectivity was found for the products **131** (Table 3).

**Table 3:** Asymmetric induction with amino acid based acylnitroso dienophiles **129**.

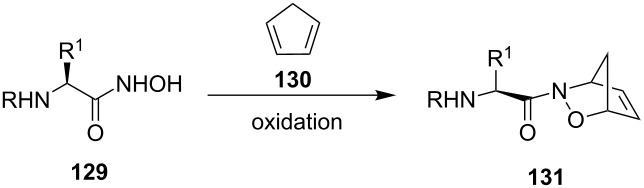			

R^a^	R^1^	Yield (%)	de (%)

Cbz or Boc	Me	90	50
Cbz or Boc	Bn	79	30
Cbz or Boc	iPr	85	60
Cbz or Boc	*t-*Bu	63	72
Cbz or Boc	Pro	75	43
Cbz or Boc	CH_2_O	77	43
Cbz or Boc	CH_2_OBn	80	38
Cbz or Boc	CH_2_-*p*-C_6_H_6_-OH	67	45
Cbz or Boc	CH_2_CO_2_CH_3_	53	0

^a^The choice of protecting group had a negligible influence on the de values and was not specified [28].

Some more extensive studies on reactions of proline or pyrrolidine-substituted acylnitroso dienophiles **133a–g** with a number of different cyclic and acyclic dienes **132** have been carried out and the corresponding hetero-Diels–Alder products **134a–g** were obtained with improved stereoselectivity (Table 4). Ghosez reported a number of reactions of pyrrolidine-substituted acylnitroso dienophiles **133a–c** that gave the corresponding hetero-Diels–Alder products with excellent stereoselectivity for a number of cyclic dienes [114–115]. Additionally, the reaction of dienophile **133a** with the acyclic 1-methoxybutadiene gave the corresponding product with comparable stereoselectivity, with a de value of more than 98%. The simplified dimethyl derivative **133f** reported by Shustov lead to the same result, indicating that larger substituents are not necessary for the stereoselectivity [116]. These results are improved relative to those initially reported by Defoin for reactions of chiral acylnitroso dienophiles **133d** and **133e** with cyclohexadiene [107] and cyclopentadiene [117]. However, it is interesting that both groups showed that the dominant diastereomer could be altered based on the stereochemistry of the attached pyrrolidine. The imidazolidin-2-one auxiliary **133g** was also used by Orena in reactions with cyclopentadiene and cyclohexadiene, giving the products with de values of 74 and 86%, respectively [118].

**Table 4:** Asymmetric induction with pyrrolidine and imidazolidin-2-one-substituted acylnitroso dienophiles (a negative de value indicates the opposite diastereomer).

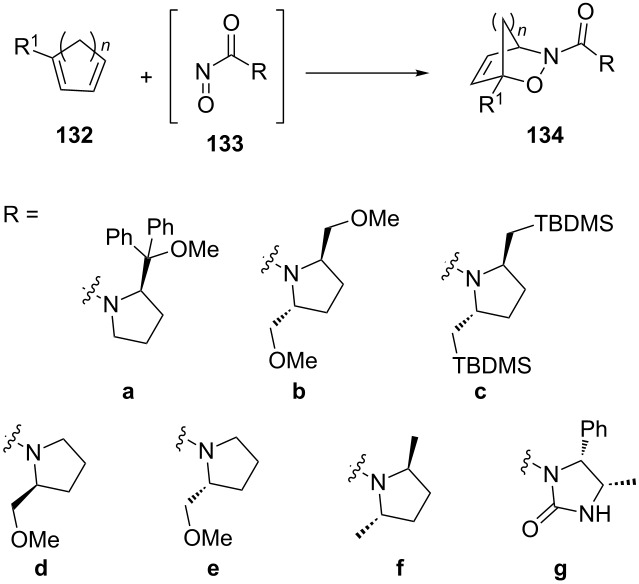				

*n*	R	R^1^	Yield (%)	de (%)

2	**a**	H	75	>98^a^
2	**a**	OMe	68	>98^a^
2	**a**	COOMe	40	>98^a^
1	**b**	H	83	87^a^
2	**b**	H	88	>98^a^
3	**b**	H	70	>98^a^
2	**c**	H	82	>98^a^
1	**d**	H	71	34^b^
2	**d**	H	80	72^c^
2	**e**	H	88	−70^c^
2	**f**	H	81	98^d^
1	**g**	H	63	74^e^
2	**g**	H	73	86^e^

^a^From reference [115]; ^b^from reference [117]; ^c^from reference [107]; ^d^from reference [116]; ^e^from reference [118].

**Scheme 26:**
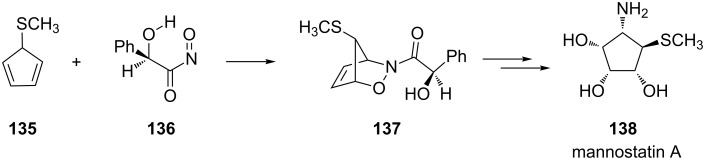
Asymmetric cycloaddition of the acylnitroso compound **136** to diene **135**.

A series of chiral auxiliaries based on mandelic acid showed similar stereoselectivity benefits and have been proposed by Procter for stereoselective synthesis [109,119]. Their utility was demonstrated by the synthesis of (+)-mannostatin A (**138**, Scheme 26) [120]. Here, the symmetric cycloaddition of the acylnitroso compound **117**, derived from (*R*)-mandelic acid, to 1-(methylthio)cyclopenta-2,4-diene (**135**) predominantly afforded the bicyclic adduct **137** (in a ratio of 3.3:1 with its diastereomer, not shown) in 45–50% overall yield.

The camphor-derived nitroso agents **140a–d** have also been used with great success in hetero-Diels–Alder reactions (Table 5). The sultam **140a** gave the products with high stereoselectivity when reacted with both cyclopentadiene and cyclohexadiene [114,121], as did the *tert*-butyl-protected camphor derivative **140b** [65]. The reaction of acylnitroso derivative **140c** with cyclopentadiene was highly stereoselective, with a de value of more than 99% for the product [122]. In case of substrate **140d**, the stereoselectivity was lower but the product was still obtained with satisfying de values of 82% for the reaction with cyclohexadiene and 84% with cycloheptatriene, respectively [123]. Dienophile **140b** was also reacted successfully with the acyclic dienes 1,4-dimethyl-1,3-butadiene and the ethyl ester of 2,4-hexenoic acid giving the products with de values of more than 95% in both cases [65].

**Table 5:** Asymmetric induction with camphor-based nitroso dienophiles, showing diastereoselectivity in the formation of derivatives **141**.

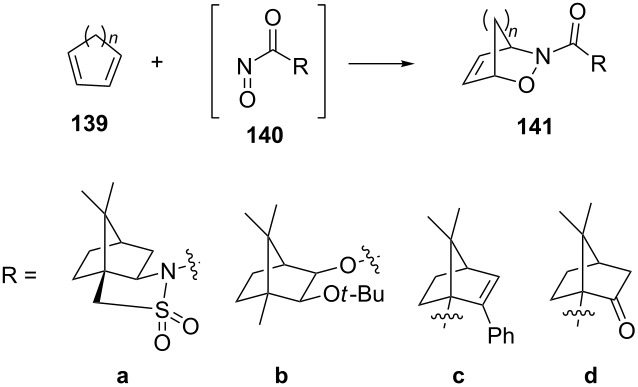			

*n*	R	Yield (%)	de (%)

1	**a**	91	>98^a^
2	**a**	94	>98^a^
1	**b**	89	91^b^
2	**b**	93	95^b^
1	**c**	94	>98^c^
2	**d**	81	82^d^
3	**d**	78	84^d^

^a^From reference [121]; ^b^from reference [65]; ^c^from reference [122]; ^d^from reference [123].

**Scheme 27:**
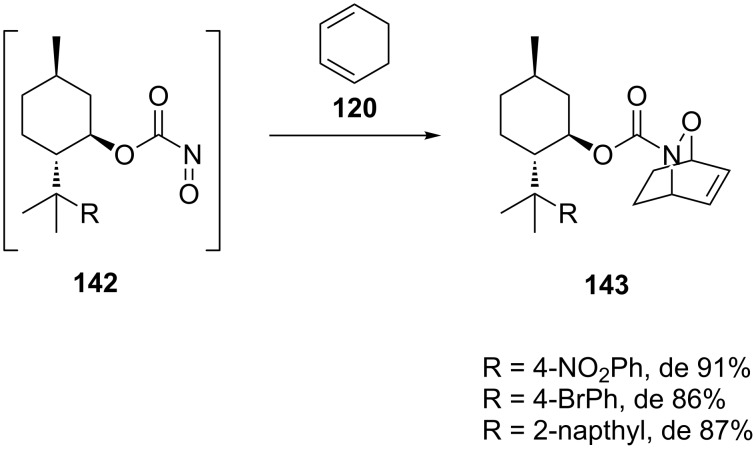
Asymmetric induction with arylmenthol-based nitroso dienophiles **142**.

Kibayashi prepared a set of optically active acylnitroso arylmenthol derivatives **142** (Scheme 27) that were subsequently reacted with 1,3-cyclohexadiene (**120**) to give the hetero-Diels–Alder products **143**, serving as intermediates for an asymmetric total synthesis of (−)-epibatidine [5,124]. The stereoselectivity of the reaction was affected by the substituent on the menthol group. The introduction of a phenyl and a 4-methoxyphenyl substituent gave the products with de values of >85%, but this could be improved to up to 91% for the 2-napthyl, 4-bromophenyl and 4-nitrophenyl substituted derivatives. All 1,2-oxazines were isolated in high yield (>94%).

**Scheme 28:**
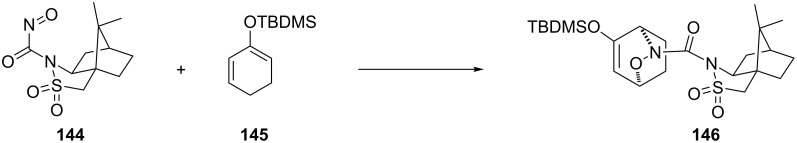
Cycloaddition of silyloxycyclohexadiene **145** to the acylnitroso dienophile derived from (+)-camphorsultam **144**.

An asymmetric hetero-Diels–Alder reaction used for the synthesis of (−)-epibatidine was reported by Royer [125–126]. The crucial step of the epibatidine synthesis, proceeding in 64% yield, is based on the asymmetric cycloaddition of the silylated diene **145** to the acylnitroso compound **144** (Scheme 28).

**Scheme 29:**
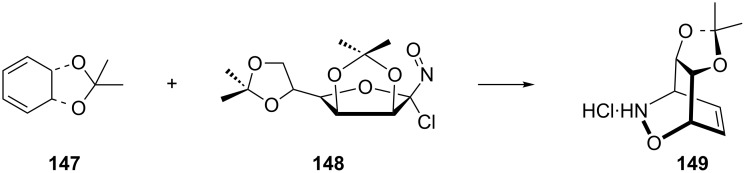
Asymmetric reaction of *O*-isopropylidene-protected *cis*-cyclohexa-3,5-diene-1,2-diol **147** with mannofuranosyl chloride **148**.

The first step of the synthesis of *cis*-1,3-diamino-1,3-dideoxycyclitols **149** starts with an asymmetric hetero-Diels–Alder reaction of *O*-isopropylidene-protected *cis*-cyclohexa-3,5-diene-1,2-diol **147** with (−)-2,3:5,6-di-*O*-isopropylidene-1-nitroso-α-D-mannofuranosyl chloride (**148**, Scheme 29) [127]. The 1,2-oxazine **149** was obtained as an optically pure (+)-*endo*-adduct.

**Scheme 30:**
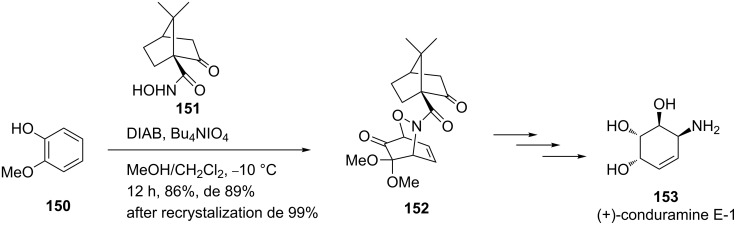
Synthesis of synthon **152** from 2-methoxyphenol **150** and chiral auxiliary **151**.

In 2010, the Liao group reported the synthesis of optically pure conduramines employing the highly stereoselective hetero-Diels–Alder reaction of nitroso dienophiles with masked *o*-benzoquinones [128]. For instance, they synthesized synthon **152** from 2-methoxyphenol (**150**) and the chiral auxiliary **151** with 99% de, which was further utilized for the synthesis of (+)-conduramine E-1 (**153**, Scheme 30).

**Scheme 31:**
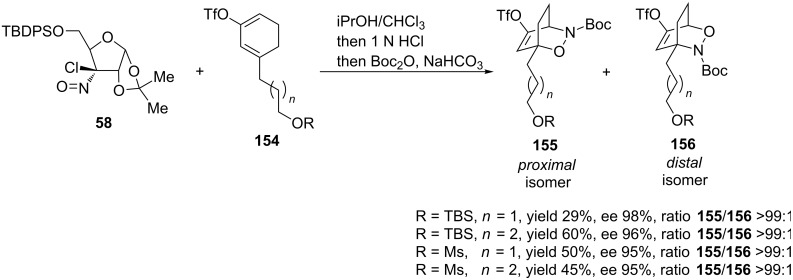
Asymmetric nitroso hetero-Diels–Alder reaction with Wightman chloronitroso reagent **58**.

The hetero-Diels–Alder reaction of chloronitroso reagent **58** with cyclohexadiene derivative **154** led to the exclusive formation of *proximal* isomers **155** in moderate to good yields with ee values ≥95% (Scheme 31). The regioselectivity was opposite of that obtained in the copper-catalyzed reaction. The authors explained this by means of the proposed transition states, where the *distal* isomer is unfavored due to steric repulsions between the side chain and the acetonide group.

**Chiral dienes:** The use of chiral cyclic and acyclic dienes for both acyl and arylnitroso dienophiles is well reported in the literature. This is in part due to the extensive use of the nitroso hetero-Diels–Alder reaction in the synthesis of many natural products. In particular, the reaction with cyclic dienes, often proceeds with very high stereoselectivity. In the review by Miller [16] the lower stereoselectivity observed for acyclic dienes was assumed to be due to the proposed asynchronous transition state of the hetero-Diels–Alder reaction in which the chiral moiety of 1-substituted dienes is spatially distant from the bulk of the incoming dienophile, due to the placement of the nitrogen substituent close to the diene.

**Scheme 32:**
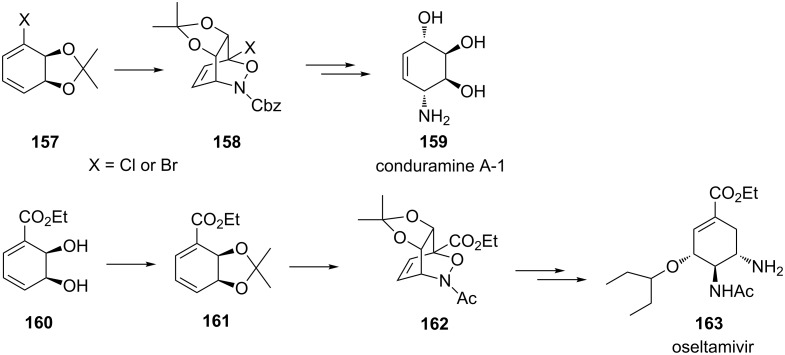
Asymmetric 1,2-oxazine synthesis using chiral cyclic diene **157** and the application of this reaction to the synthesis of conduramine A-1 **159** and oseltamivir **163**.

Hudlicky and co-workers extensively used chiral cyclic dienes obtained by microbial oxidation of halobenzenes for the preparation of nitroso hetero-Diels–Alder products. In the synthesis of conduramine A-1, nitroso hetero-Diels–Alder products **158** obtained from chiral dienes **157** were formed diastereoselectively in moderate yields with no evidence of either diastereomeric or regioisomeric contaminants (52–54%, Scheme 32) [129]. These reactions have subsequently been applied to the synthesis of oseltamivir (Tamiflu^TM^), where the yield of the Diels–Alder product **162** were considerable higher (70% from **160**, Scheme 32) [7].

**Scheme 33:**
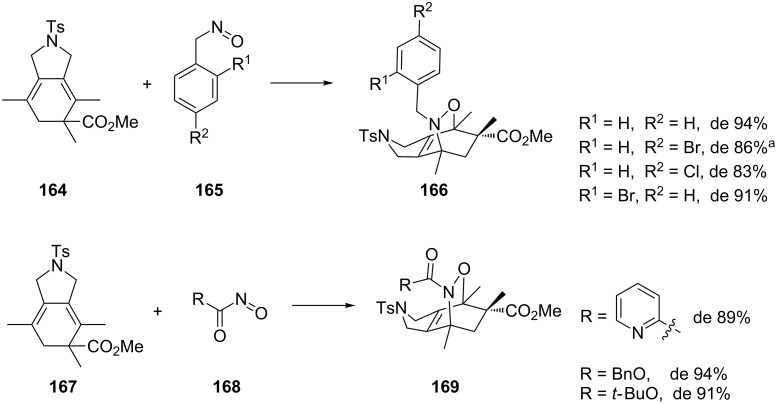
Asymmetric 1,2-oxazine synthesis using a chiral diene reported by Jones et al. [75]. ^a^Regioisomeric ratio 3.5:1.

Another chiral diene **164** was used by Jones in the reaction with a number of achiral alkyl **165** and acylnitroso dienophiles **168** (Scheme 33) [75]. The reactions were regioselective for the 1,2-oxazines in most cases and good stereoselectivities were achieved, with de values of up to 94%.

**Scheme 34:**
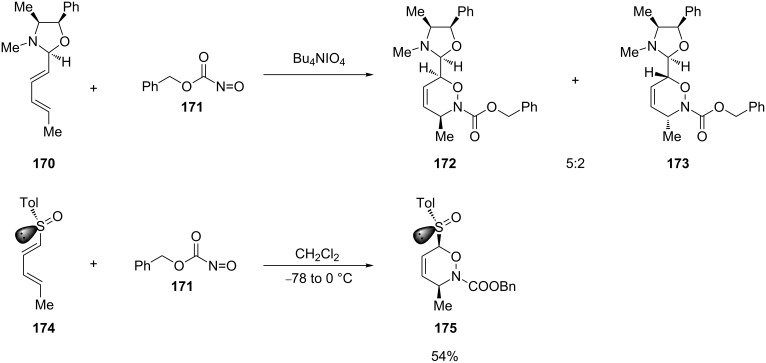
The nitroso hetero-Diels–Alder reaction of acyclic oxazolidine-substituted diene **170** and chiral 1-sulfinyl diene **174** with benzyl nitrosoformate.

Even though the use of acyclic dienes generally affords 1,2-oxazines with lower diastereoselectivities than with the corresponding cyclic counterparts, they have been successfully applied in nitroso hetero-Diels–Alder reactions. For example, the diene substituted with a pseudoephedrine-derived oxazolidine **170** gave the product in only modest stereoselectivity when reacted with benzyl nitrosoformate (**171**) [130]. On the other hand, the reaction of the chiral 1-sulfinyl diene **174** was found to be completely regio- and stereoselective when reacted with the same dienophile (Scheme 34) [131–132].

**Scheme 35:**
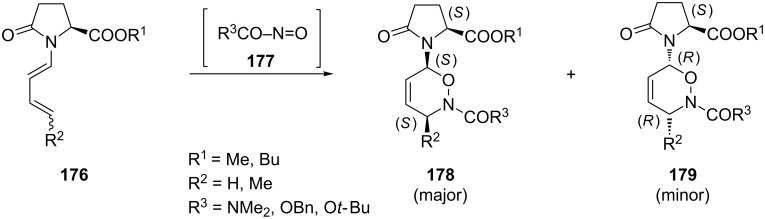
The nitroso hetero-Diels–Alder reaction of acyclic lactam-substituted diene **176** with various acylnitroso dienophiles **177**.

The acyclic lactam-substituted diene **176** was reacted with a range of acylnitroso dienophiles **177** and the corresponding products were obtained with de values of up to 90% (Scheme 35) [133–134].

#### Stereoselective control of the nitroso hetero-Diels–Alder reaction by catalysis

The highly interesting products that are accessible through the nitroso hetero-Diels–Alder reaction has led to the development of a catalytic version of this cycloaddition. In principle, there are two conceptually different approaches for the catalytic hetero-Diels–Alder reaction reported in the literature [135], which will be exemplified below.

**Scheme 36:**
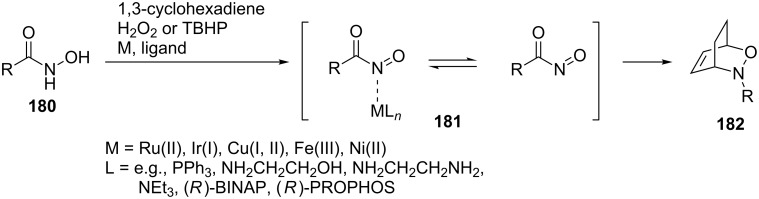
The hetero-Diels–Alder reaction of acylnitroso dienophile.

**Scheme 37:**
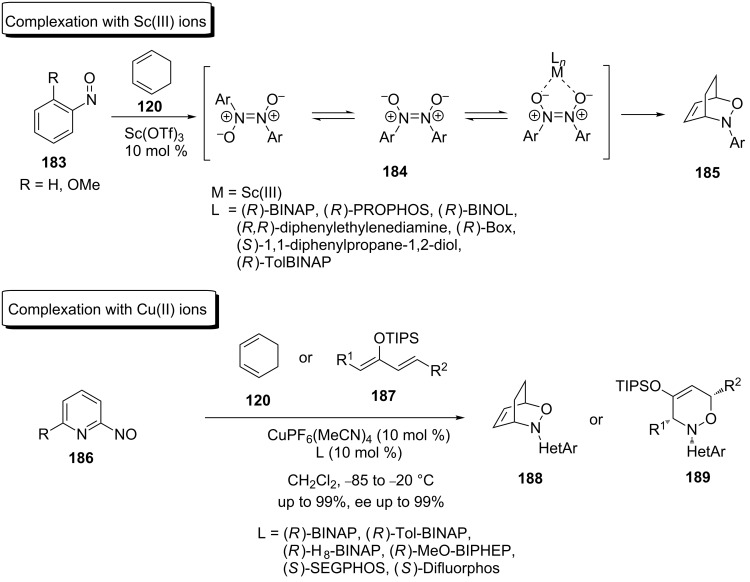
The hetero-Diels–Alder reaction of arylnitroso dienophiles using Lewis acids.

a) The first approach is based on the in situ generation and trapping of an intermediate acylnitroso dienophile in the presence of transition metals (Scheme 36), e.g., [67,136–141].

b) The second approach relies on the activation of a moderately reactive arylnitroso dienophile by scandium [142] or copper [103–104] metal ions, which are able to complex a nitroso derivative with chiral ligands (Scheme 37).

**Scheme 38:**
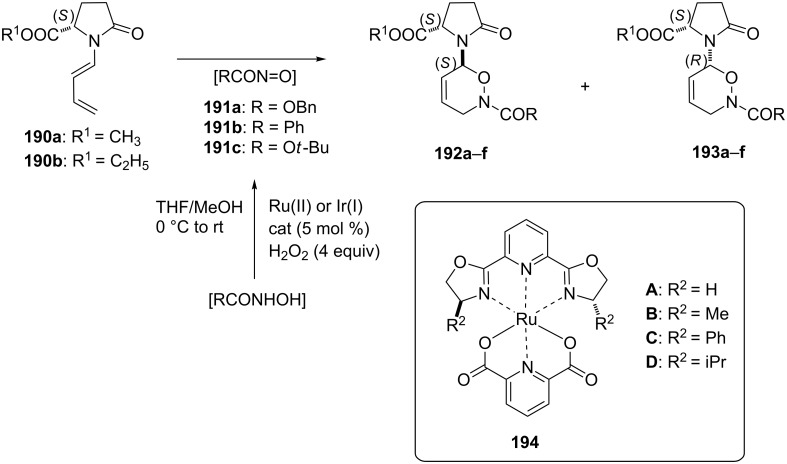
Asymmetric hetero-Diels–Alder reactions of chiral alkyl *N*-dienylpyroglutamates.

**Approach based on the in situ generation and trapping of an intermediate acylnitroso dienophile:** This approach is exemplified by the results of the Iwasa group from 2008. They performed asymmetric hetero-Diels–Alder reactions of chiral alkyl *N*-dienylpyroglutamates **190** with acylnitroso intermediates **191** generated through a Ru(II) or Ir(I)-catalyzed hydrogen peroxide oxidation of hydroxamic acids (Scheme 38) [143]. The Ru(II) complexes **A**–**D** have previously been reported [144] as efficient catalysts for the oxidation of hydroxamic acids and therefore they were applied to the synthesis of transient acylnitroso intermediates. Moderate to high yields (45–98%) with good diastereoselectivities (de 42–72%) were obtained through the hetero-Diels–Alder reaction of chiral lactams with acylnitroso intermediates.

**Scheme 39:**
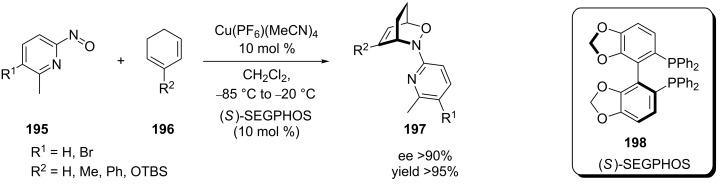
Catalytic asymmetric arylnitroso reaction between mono-substituted 1,3-cyclohexadiene **196** and disubstituted nitrosopyridine **195**.

**Approach based on the activation of a moderately reactive arylnitroso dienophile; coordination of the nitroso dienophile:** The effective catalytic asymmetric nitroso hetero-Diels–Alder reaction is a relatively new method, first reported within the last decade. Before this time, only very low ee values (less than 15%) were obtained when the reaction was performed in the presence of Lewis acids [142]. In 2004, the Yamamoto group reported results from an asymmetric nitroso hetero-Diels–Alder reaction between 6-substituted nitrosopyridine **195** (where R^1^ = H) and 1,3-cyclohexadienes **196** using Cu(PF_6_)(MeCN)_4_-(*S*)-BINAP (**198**) as the catalyst (Scheme 39) [104]. The reaction was highly sensitive to the substituent R^2^ on the dienophile, with the methyl group giving the best stereoselectivity (87% relative to 56%, when R^2^ = H). After testing several BINAP-like catalysts, the reaction of the unsubstituted and monosubstituted 1,3-cyclohexadiene **196** with the substituted pyridylnitroso species **195** with (*S*)-SEGPHOS (**198**) as the chiral ligand gave the best results. An excellent regioselectivity towards distal isomer **197** was observed in each applicable case and the product was obtained in good yield and with ee values higher than 90% (Scheme 39).

However, the application of this reaction to acyclic dienes was not successful; poor regio- and stereoselectivities were obtained for the reaction of 1,3-pentadiene **200** and 6-methyl-2-nitrosopyridine (**199**). After enhancing the reactivity of the diene by using the 3-trimethylsiloxy-substituted 2,4-hexadiene the regioselectivity of the reaction was increased, but the stereoselectivity remained low. Exchanging the siloxy substituent (TBS) by the bulkier TIPSO group greatly increased the stereoselectivity and the product was obtained with an ee value of 98%. Further it was shown that (*S*)-Difluorphos **202** gave complete stereoselectivity (ee: >99%) for the hetero-Diels–Alder product **201**. This catalyst system was then applied to a number of substituted dienes **200** and excellent regio- and stereoselectivities were obtained for the products (Table 6) [103].

**Table 6:** Catalytic asymmetric arylnitroso hetero-Diels–Alder reaction between disubstituted acyclic dienes **200** and 6-methyl-2-nitrosopyridine (**199**).

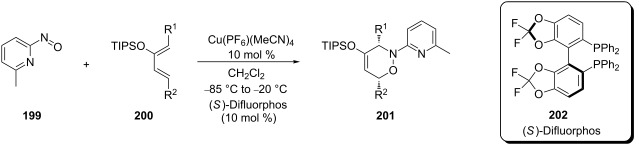			

R^1^	R^2^	Yield (%)	ee (%)

CH_3_	CH_3_	95	99
CH_3_	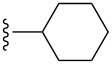	93	91
CH_3_	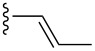	91	96
CH_3_	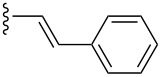	84	85
CH_3_	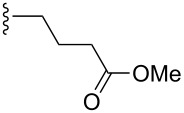	96	93
CH_3_	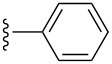	95	81
CH_3_	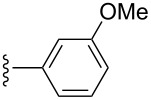	91	99
CH_3_	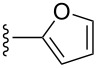	91	95
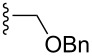	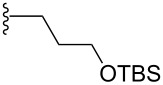	86	95
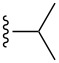	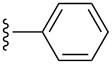	97	95
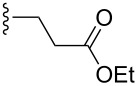	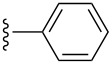	94	88

**Figure 7:**
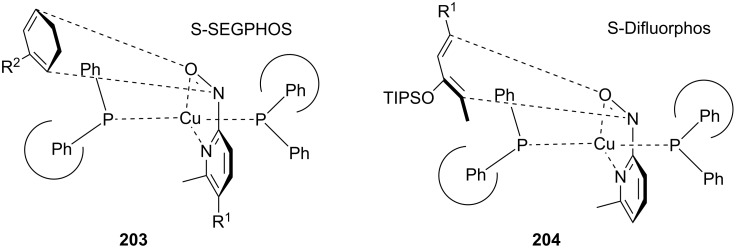
Plausible chelate intermediate complexes formed during the hetero-Diels–Alder reaction to give 1,2-oxazines **203** and **204**.

The observed stereoselectivity of this reaction may be explained by the coordination of both the nitroso dienophile and the nitrogen on the pyridine ring to the Cu(I) center of the catalytic complex. Using a chiral ligand with a narrow dihedral angle [145–146] gives a highly organized transition state with a clearly defined approach for the diene to interact with the dienophile. Similar plausible chelate intermediates were proposed for each example (Figure 7) and they were proven to match that found for the reaction products [103–104]. The sensitivity of the cyclic dienes to the 6-substituent on the dienophile and the dihedral angle of the chiral ligand are consistent with this proposed complex and show that it is a highly ordered chelate intermediate. The authors suggested that the size of the TIPS group forces the diene into an *s*-*cis* conformation, which promotes the [4 + 2] concerted cycloaddition reaction.

In 2007, Studer reported on the results of the kinetic resolution of racemic dienes by subjecting them to a Cu(I)-catalyzed nitroso hetero-Diels–Alder reaction in the presence of chiral diphosphine ligands [147]. The best results were obtained using Walphos ligand **209** and the results of the reactions of dienes **205** with 2-nitrosopyridine (**206**) are collected in Table 7.

**Table 7:** Summary of the catalytic kinetic resolution of substituted cyclohexadienes **205** through a nitroso hetero-Diels–Alder reaction.

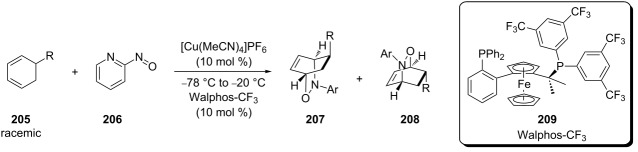				

R	**207**		**208**	

	ee (%)	Yield (%)	ee (%)	Yield (%)

CMe_2_OTMS	95	48	89	52
CH_2_OTBDMS	99	42	88	45
CH_2_Ph	98	40	84	43
CH_2_OMe	98	39	82	42
Ph	98	45	94	54

**Scheme 40:**
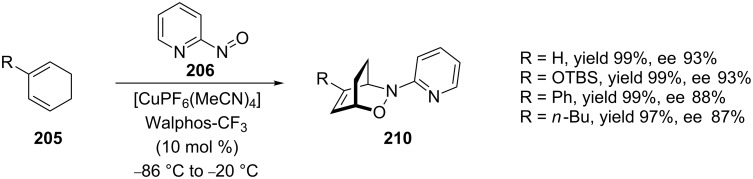
Catalytic asymmetric nitroso hetero-Diels–Alder between cyclic dienes and 2-nitrosopyridine.

This method was subsequently applied to the first total synthesis of enantiomerically pure (+)-*trans*-dihydronarciclasine [3] and, with a number of cyclic dienes, **205** gave ee values of 81–95% for hetero-Diels–Alder products **210**, some of which are shown in Scheme 40 [148]. The result for the reaction of 1,3-cyclohexadiene with the unsubstituted pyridyl dienophile (ee 93%) was equal to that reported by Yamamoto for the 2-methyl-substituted pyridyl dienophile (92%) [104].

**Scheme 41:**
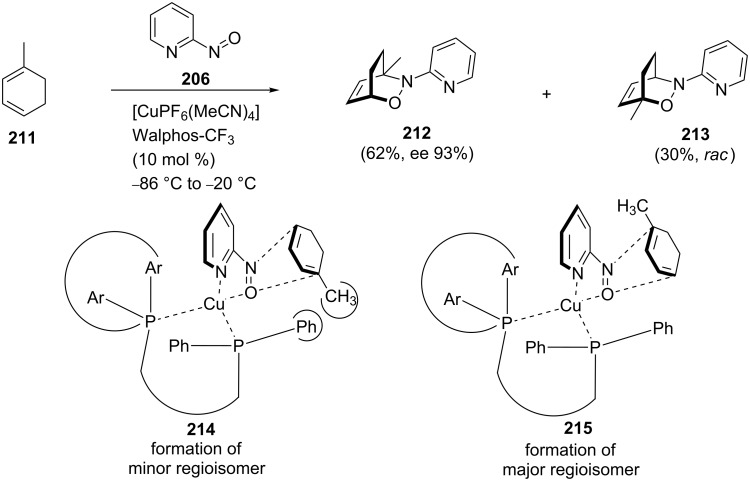
The reason for the increased enantioselectivity of stereoisomer **212** compared with stereoisomer **213**.

When the diene 1-methylcyclohexadiene (**211**) was subjected to the above conditions, the regioselectivity was found to be low, compared to the other examples. The *proximal* (minor) regioisomer **213** was isolated as a racemic mixture, but the *distal* (major) regioisomer **212** showed good stereoselectivity (Scheme 41). Subsequently, the complex of the Cu(I)/Walphos-CF_3_ catalyst system with nitrosopyridines **214** and **215** was studied theoretically using DFT calculations to determine the structure (Scheme 41). As previously discussed, calculations by Houk indicated that the diene will preferentially attack through the *endo* pathway [79–80], and, as shown, this would result in one substituent on the cyclohexadiene sterically clashing with a phenyl group of the Walphos-CF_3_ catalyst. This is probably the reason why the regioselectivity for diene **211** was low, and the stereoselectivity for the *proximal* isomer was poor, as the cyclization likely proceeded without the involvement of the catalyst [148].

Interestingly, although the ee values were very similar for the reaction of 1,3-cyclohexadiene with the pyridyl dienophile using the Walphos-CF_3_ catalyst (>87%) and (*S*)-Difluorphos (>85%), they have differing stereoselectivities.

**Scheme 42:**
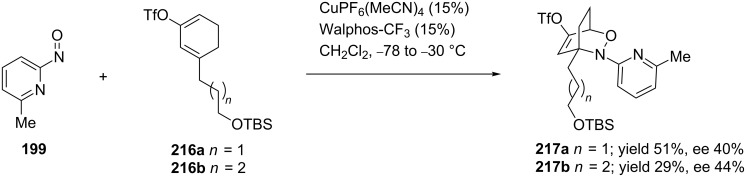
The copper-catalyzed nitroso hetero-Diels–Alder reaction of 6-methyl-2-nitrosopyridine (**199**) with protected dienes **216** with Walphos-CF_3_ as the ligand.

On the other hand, when Kouklovsky performed the copper/Walphos-CF_3_-catalyzed nitroso hetero-Diels–Alder reaction with 6-methyl-2-nitrosopyridine (**199**) and the protected dienes **216** (Scheme 42) [90], the cycloadducts **217** (with the exclusive formation of *distal* isomers) were obtained with strongly diminished yields and enantioselectivities. For this reason, the authors selected another method for the asymmetric nitroso hetero-Diels–Alder reaction, using the Wightman chloronitroso reagent **58** as a chiral dienophile (Scheme 31).

**Scheme 43:**
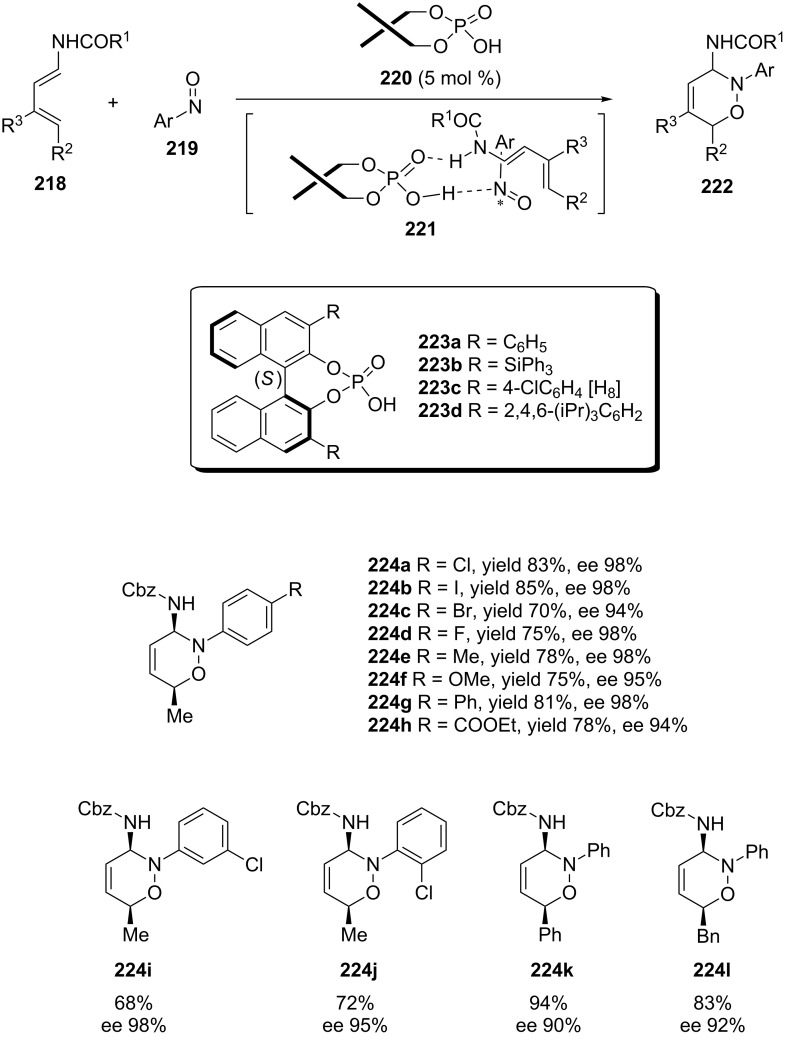
Asymmetric nitroso hetero-Diels–Alder reaction of nitrosoarenes with dienylcarbamates catalyzed by chiral phosphoric acids.

In 2015, Masson published a chiral phosphoric acid-catalyzed asymmetric nitroso hetero-Diels–Alder reaction of nitrosoarenes with substituted dienylcarbamates (Scheme 43) [149]. The reaction afforded the *cis*-3,6-disubstituted dihydro-1,2-oxazines **222** in high yields with excellent regio-, diastereo- and enantioselectivities. These cycloaddition conditions are applicable to a wide range of nitrosoaryl derivatives and dienylcarbamates and some representative examples are depicted in Scheme 43.

**Scheme 44:**
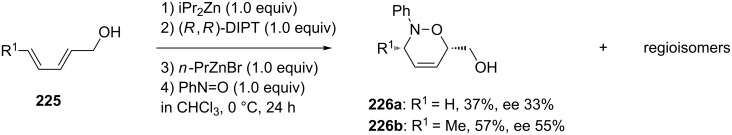
The enantioselective hetero-Diels–Alder reaction between nitrosobenzene and (*E*)-2,4-pentadien-1-ol (**225**) in the presence of tartaric acid ester.

**Approach based on the activation of a moderately reactive arylnitroso dienophile; coordination of the diene:** The Inomata group studied a different system utilizing the coordination of a hydroxylated diene to a tartaric acid ester [150–152]. When they examined the enantioselective hetero-Diels–Alder reaction between nitrosobenzene and (*E*)-2,4-pentadien-1-ol (**225**) (Scheme 44), they obtained the corresponding dihydro-1,2-oxazine **226** as a mixture of regioisomers. The ee values for the major products **226a** and **226b** were 33% and 55%, respectively. This modest enantioselectivity encouraged the Inomata group to further investigate the reaction [152].

**Scheme 45:**
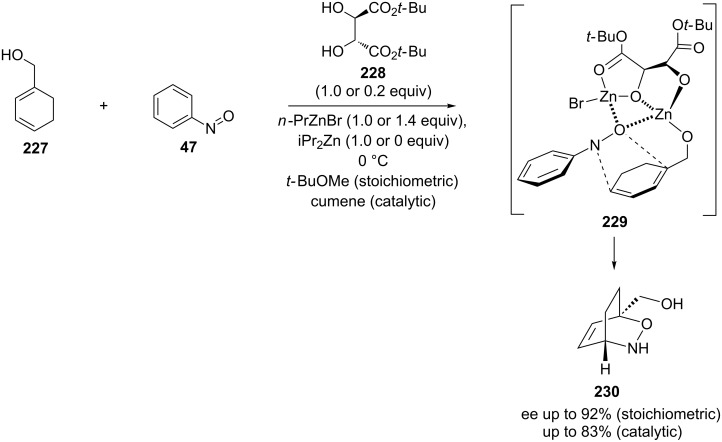
Asymmetric nitroso hetero-Diels–Alder reaction using tartaric acid ester chelation of the diene and dienophile with the proposed chelate complex.

Their investigation of enantioselective hetero-Diels–Alder reactions resulted in an enantioselectivity of up to 92% ee, when nitrosobenzene and a dienol were reacted in the presence of tartaric acid ester as a chiral auxiliary. Moreover, the reaction was completely regioselective [151]. In further experiments, one equivalent of *n*-propylzinc bromide, diisopropylzinc, (*R*,*R*)-diisopropyl tartrate and the diene were mixed to form a complex before the nitroso dienophile was added. In case of the acyclic dienes tested, no regioselectivity was observed, and the products were obtained with poor stereoselectivity (ee 33–55%). However, with cyclic diene **227**, where the diisopropyl tartrate ester was replaced with the bulkier *tert*-butyl ester **228** and the dienophile **47** was added slowly over three hours, the reaction was completely regioselective, and good stereoselectivity was observed for the hetero-Diels–Alder product **230** (ee values up to 92%) (Scheme 45) [150]. A coordination complex **229** was proposed as source of the regio- and stereoselectivity in which both, the diene and the dienophile, are bound to the tartrate ester. The same reaction carried out using a catalytic amount (20 mol %) of the di-*tert*-butyl tartrate and 1.4 equiv of *n*-propylzinc bromide resulted in an enantiomeric excess of up to 83% for the product. It should be noted that 4 Å molecular sieves, to provide extremely anhydrous conditions, were vital for the reproducibility of the regio- and stereoselectivity for this catalytic reaction.

## Conclusion

Nitroso hetero-Diels–Alder reactions have been used successfully for the synthesis of many biologically active molecules. The success of this methodology may be explained in part by the high selectivity of this reaction. The resulting 3,6-dihydro-2*H*-1,2-oxazine scaffold, facilitates a plethora of subsequent transformations towards various derivatives. The 1,2-oxazines are the result of what can be considered an extraordinarily mild and effective 1,4-aminohydroxylation reaction. The observed high regioselectivity results from various electronic effects and the stereoselectivity can be influenced by the use of chiral dienes or dienophiles or the application of asymmetric catalysis. These methods, mainly the last one, take place in solution, while the solid-phase approaches described in the literature were not studied in view of stereoselectivity at all. The regiocontrol of hetero-Diels–Alder reactions requires additional research for both, solution as well as solid-phase synthesis. The regiocontrol in solution involving arylnitroso derivatives has been poorly explored, while the use of catalysts with acylnitroso compounds is still lacking in the literature. Reactions on solid supports are described in a few articles as being regioselective, but these results focus on the nature of reagents, while regiocontrol via external assistance is mentioned only once. The stereo- and regioselective hetero-Diels–Alder reaction should be more closely studied to apply and extend the methods discussed here providing 1,2-oxazine products that can be utilized for the synthesis of important molecules.
